# Characterising the Antimicrobial Performance of Engineered Layered Double Hydroxide Surfaces for Biofilm Control

**DOI:** 10.3390/nano16110666

**Published:** 2026-05-25

**Authors:** Federico Delle Fave, Michela Froio, Diego Cisternino, Suguna Jayaraman, Chris Ashley, Pier Gianni Medaglia, Francesco Giorgi

**Affiliations:** 1 Department of Enterprise Engineering, University of Rome Tor Vergata, 00133 Roma, Italy; federico.dellefave@alumni.uniroma2.eu (F.D.F.);; 2 Department of Material Design and Manufacturing Engineering, School of Engineering, University of Liverpool, Liverpool L69 7ZX, UKc.ashley3@liverpool.ac.uk (C.A.); francesco.giorgi@liverpool.ac.uk (F.G.); 3 Department of Industrial Engineering, University of Rome Tor Vergata, 00133 Roma, Italy

**Keywords:** layered double hydroxides, antimicrobial surfaces, nanostructure, biofilm, antimicrobial resistance

## Abstract

Antimicrobial resistance (AMR) is a growing global health concern driven by bacterial biofilm formation, which increases tolerance to treatments. Developing surface-based strategies to limit biofilm formation is therefore critical. Layered Double Hydroxides (LDHs) are 2D brucite-like nanomaterials with tuneable physicochemical properties that may reduce bacterial colonisation. Their ease of synthesis, with scalability potential for industrial production, alongside their characteristic and tunable physicochemical properties, makes them a promising nanostructured coating for antimicrobial applications. This study evaluates LDH thin-film coatings as intrinsic antimicrobial surfaces, focusing on the combined effects of chemical composition, nanotopography, and wettability on biofilm formation in *Escherichia coli*, *Staphylococcus aureus*, and *Pseudomonas aeruginosa*. Four aluminium-based LDHs (ZnAl-NO_3_, ZnAl-Cl_2_, MgAl-NO_3_, MgAl-Cl_2_) were synthesised via coprecipitation or in situ growth on aluminium substrates. Materials were characterised by XRD, SEM, EDS, and contact angle measurements. Antimicrobial performance was assessed by quantifying colony-forming units (CFU mL^−1^) after bacterial exposure. ZnAl-LDH surfaces showed significant antimicrobial activity against *E. coli* and *S. aureus*, while MgAl-LDHs showed no effect and occasionally increased bacterial growth. None of the LDH surfaces tested exhibited significant antimicrobial activity against *P. aeruginosa* strain. The antimicrobial performance of ZnAl-LDH can be attributed to the concurrent effect of the surface chemistry, wettability, and sharp platelet-like nanotopography. The results obtained demonstrate that ZnAl-LDH-based coatings are promising antimicrobial materials with potential relevance for translational research in clinical antimicrobial surface development.

## 1. Introduction

Antimicrobial resistance (AMR) has emerged as a critical global public health threat. In 2019 alone, AMR was directly responsible for an estimated 1.27 million deaths and contributed to nearly 5 million deaths worldwide [1], resulting in healthcare losses exceeding €11.7 billion annually within the European Union health systems [2]. If the current trajectory remains unchecked, the global economic burden of AMR could reach up to US$100 trillion by 2050 [2,3].

AMR arises when bacterial populations acquire or develop mechanisms that enable survival in the presence of antimicrobial agents designed to eliminate them. One of the most significant contributors to bacterial persistence is the formation of biofilms, structured microbial communities adhering to biotic or abiotic surfaces and encased within a self-produced extracellular polymeric matrix. In this state, bacteria can exhibit up to 1000-fold higher tolerance to antibiotics compared with planktonic (free-floating) cells [4]. This increased tolerance primarily results from limited antibiotic penetration, reduced metabolic activity within the biofilm, and the induction of specific resistance genes [4]. Biofilm-associated infections are frequently observed in chronic wounds, urinary tract infections, and on contaminated medical devices [4]. Because conventional antimicrobials and chemical disinfectants are markedly less effective against biofilms, novel non-antibiotic strategies, particularly those that prevent initial bacterial adhesion, colonisation and biofilm formation on surfaces, are urgently needed.

**Figure 1:**
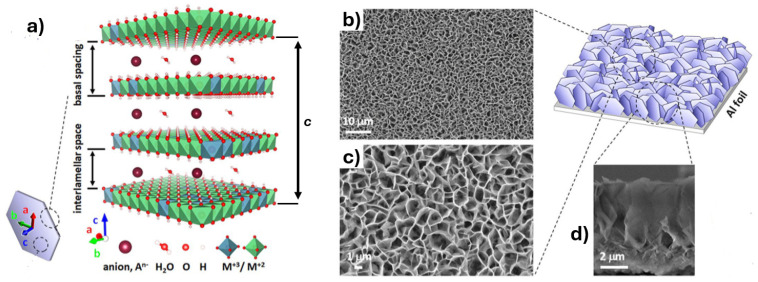
(**a**) Schematic illustration of the crystalline structure of layered double hydroxides (LDHs); (**b**) SEM image of the LDH surface (top view) at low magnification; (**c**) SEM image of the LDH surface (top view) at high magnification; (**d**) SEM image of the LDH cross-section (side view, 2 μm scale). Adapted from [30].

Layered double hydroxides (LDHs) are a class of two-dimensional anionic clays consisting of positively charged brucite-like layers formed by metallic cations (generally divalent or trivalent) octahedrally coordinated with 6 OH^−^ groups (Figure 1a). These layers, called lamellas, are intercalated with charge-balancing anions and water molecules [5,6] in the so-called interlamellar spaces. Their general formula is:

[M2+(1−x)M3+(x)(OH)2]x+(An−)xn·yH2O

where M^2+^ and M^3+^ denote the divalent and trivalent metal cations, such as Mg^2+^, Zn^2+^ or Al^3+^, Fe^3+^, and A^n−^ represents the interlayer anion. Amongst the family of LDH, Mg^2+^ and Zn^2+^ are by far the most used divalent cations as they ensure greater stability of octahedral arrangement in the brucite-like sheets. At the same time, among the substituent trivalent cations, Al^3+^ is the most commonly chosen due to its small ionic radius (close to those of Mg^2+^ and Zn^2+^) able to preserve stability and high degree of crystallinity of hexagonal lattice. Furthermore, aluminium is environmentally friendly, non-toxic and cost-effective. Finally, Al^3+^ is preferable in comparison to cobalt and iron, which can easily change the oxidation state and require complex synthesis routes to control stoichiometry. At the nanoscale, LDHs typically exhibit leaf-like morphologies of interconnected nanoplatelets (Figure 1), with high aspect ratios and tuneable lateral dimensions. This architecture confers a combination of properties that are particularly relevant for advanced applications as the interlayer space allows the exchange and intercalation of a wide variety of anionic species, while the high surface area and charge density enhance adsorption and catalytic behaviour. Their layered architecture, tuneable chemistry, and controlled release properties make LDH nanostructures attractive for environmental remediation [7], e.g., as adsorbents for heavy metals and organic pollutants, and for applications in catalysis and energy storage [8]. Many LDH formulations are also stable under physiological conditions and display low cytotoxicity, which has stimulated interest in their use for biomedical purposes [9], gas- and bio- sensors [10,11,12], or other bio-inspired applications [13].

Specifically, LDH nanostructures in particulate form have been proven to act as intrinsic antimicrobials as well as carriers for bioactive ions or drugs. For example, green-synthesised Cu–Al LDH nanoparticles modified with plant extract exhibited antibacterial activity against both Gram-positive and Gram-negative bacteria while maintaining low cytotoxicity, underscoring their potential as stand-alone antimicrobial agents or multifunctional carriers [14]. In parallel, LDHs have been incorporated into surface and film systems to create active antimicrobial coatings. Ag@NiZnAl-LDH embedded in polyethylene films, for instance, produced strong inhibition of *Staphylococcus aureus*, *Escherichia coli* and *Aspergillus brasiliensis* while showing minimal ion migration into dairy simulants, providing a practical route to active food-contact materials [15]. Moreover, composite coatings combining plasma-electrolytic oxidation with Mg–Zn–Al LDH layers on magnesium alloys have been proven to function as controlled Zn/Mg ion-release platforms to improve corrosion resistance, stimulate osteogenesis and inhibit *Staphylococcus aureus* growth, highlighting an implant-relevant antimicrobial surface strategy [16]. LDHs have also been used to host antimicrobial additives such as potassium sorbate on polypropylene, creating controlled-release surfaces for food packaging [17].

Pristine, not functionalised Al-based LDH in the form of powders have also been investigated in recent years, exhibiting antimicrobial potential thanks to their ability to interact and damage bacterial cell wall [18,19,20], release structure-forming divalent ions [21,22,23] and generate reactive oxygen species (ROS) [24]. Amongst the potential formulations, Zn-based LDHs seem to be the most efficient by either divalent ion release or cell wall binding, hindering bacterial proliferation and nutrient intake [19,21,22,23,25], with other formulations including Mg-Cu still offering considerable antimicrobial performance [24,26,27]. Despite these promising findings, most studies on pristine Al-based LDHs have focused on powders or dispersed nanoparticles synthesised by co-precipitation, with antimicrobial performance commonly assessed using inhibition-zone assays [18,19,21,22,23,24,25,26,27]. While informative, these approaches provide limited insight into how pristine LDHs behave when engineered as fixed surface nanostructures, particularly under conditions relevant to bacterial adhesion and biofilm formation. Therefore, moving beyond powder-based systems and simple planktonic or diffusion-based assays is an important step towards defining the antimicrobial performance of pristine Al-based LDH surfaces and understanding their interactions with different bacterial strains. In this context, the aim of this study is to explore the antimicrobial potential of novel, in-house synthesised Al-based LDH surfaces. Rather than incorporating external antimicrobial agents or additives, this work investigates LDHs as passive nanostructured surfaces designed to influence bacterial adhesion, survival, and early biofilm development through their intrinsic physicochemical properties. The focus on Al-based LDH 2D nanostructures investigated in this study has been primarily driven by both their promising antimicrobial performance reported in the literature and the accessibility of their synthesis: the required materials, mainly precursor metal salts, are inexpensive and non-toxic, and the equipment needed is minimal and non-specialised (e.g., oven and hot plate). In addition, surface topography influences the choice of LDH formulation, as Al-based LDHs exhibit a sharper, more spiked morphology, which may contribute to their antimicrobial effect [28,29].

This strategy advances the understanding of contact-based antimicrobial mechanisms in LDHs and outlines a pathway toward economical and scalable nanocoatings. The fabrication of these LDH surfaces requires fewer processing steps, avoids the use of expensive bioactive cargoes, and provides a stable, long-lasting antimicrobial functionality suitable for industrial and biomedical applications.

## 2. Materials and Methods

### 2.1. LDH Coprecipitation Synthesis (Etched-Glass LDH)

MgAl–NO_3_ LDH, MgAl–Cl_2_ LDH, ZnAl–NO_3_ LDH, and ZnAl–Cl_2_ LDH formulations synthesised using both coprecipitation and in situ methods. The synthesis of both coprecipitated LDH and in situ LDH followed protocol found in relevant literature [28,29,31].

For the coprecipitation method, aluminium nitrate nonahydrate (Sigma-Aldrich, St. Louis, MO, USA) was used as the aluminium source for nitrate-based LDHs, while aluminium chloride hexahydrate (Sigma-Aldrich) was used for chloride-based LDHs. The corresponding metal precursors were magnesium nitrate hexahydrate, magnesium chloride hexahydrate, zinc nitrate hexahydrate, and zinc chloride (Sigma-Aldrich, St. Louis, MO, USA) [29,31,32,33]. LDHs were synthesised by maintaining a 2:1 molar ratio between the divalent metal precursor (Mg^2+^ or Zn^2+^) and the aluminium precursor. The metal and aluminium salts were dissolved in deionised (DI) water under stirring until complete dissolution. A 1 M NaOH solution was then added dropwise to adjust the pH of the reaction mixture to 10–11. Coprecipitation was carried out at 70 °C under continuous stirring for 24 h. The resulting LDH precipitate was washed with DI water, centrifuged three times to remove residual salts and allowed to dry at 60 °C for 24 h to obtain a dry powder. The LDH suspensions were prepared by dispersing 100 mg of LDH powder in 10 mL of DI water, followed by overnight stirring at ambient temperature prior to use.

The substrate has been prepared by cutting standard glass microscopy slide into 22 mm × 22 mm squares using a diamond tip. Chemical etching was employed to introduce controlled surface roughness and increase surface energy, thereby enhancing LDH adhesion, coating uniformity, and mechanical stability during subsequent handling and bacterial assays. Etching also provides a reproducible surface topography, allowing the influence of LDH deposition to be assessed independently of uncontrolled substrate variability.

**Figure 2:**
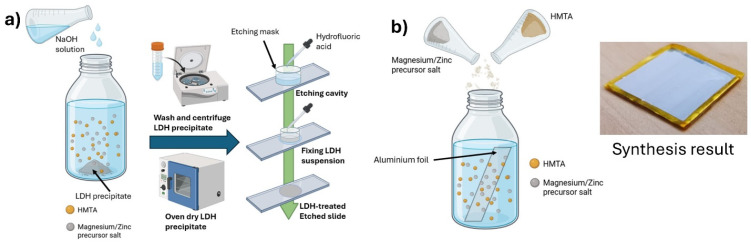
Synthesis process of LDHs; (**a**) coprecipitation of LDH,s glass etching and LDH fixing process; (**b**) in situ synthesis process with image of resulting LDH sample.

Chemical etching of the square slides was performed using 37% hydrofluoric acid (Sigma-Aldrich, St. Louis, MO, USA). The slides were placed in a custom 3D-printed mask containing a square slot of 22 mm × 22 mm and circular apertures (1.2 cm diameter), defining the active surface area. Hydrofluoric acid was added to the apertures until filled and left to etch the glass surface for 20 min (Figure 2a). Following etching, the slides and mask were thoroughly rinsed with deionised (DI) water to remove residual acid.

The etched slides were then remounted in the mask, and 150 µL of LDH suspension was deposited into each circular region. Samples were dried at 60 °C until complete solvent evaporation. Subsequently, the slides were removed from the mask and annealed at 300 °C for 12 h to improve coating consolidation and adhesion.

For comparison, etched slides without LDH deposition (“etched blank” samples) were prepared using the same protocol and used as controls for antimicrobial activity, enabling the antimicrobial effect of hydrofluoric acid etching alone to be distinguished from that of LDH treatment.

### 2.2. LDH In Situ Synthesis (In Situ LDH)

For the in situ synthesised LDH samples, an aqueous solution containing 20 mM hexamethylenetetramine (HMTA; Sigma-Aldrich, St. Louis, MO, USA) and 20 mM of the relevant metal precursor salt (magnesium nitrate hexahydrate, magnesium chloride hexahydrate, zinc nitrate hexahydrate, or zinc chloride; Sigma-Aldrich, St. Louis, MO, USA) was prepared in deionised (DI) water. An 80 µm-thick aluminium foil (Aluxfoil, Budapest, Hungary), acting as the aluminium source, was secured onto a 22 mm × 22 mm glass coverslip, then secured onto a glass slide using Kapton tape and submerged in the reaction solution.

The glass slide was positioned such that the aluminium foil was oriented at approximately 45° relative to the horizontal plane and facing the bottom of the reactor (Figure 2b). This configuration was selected as it minimises unwanted side reactions and suppresses the formation of secondary phases, such as zinc oxide, on the aluminium surface, thereby promoting controlled LDH growth [32]. The reaction vessel was then placed in an oven at 70 °C to initiate in situ LDH formation. Reaction time depended on the specific LDH composition, specifically 6 h for ZnAl–NO_3_ LDH, 24 h for MgAl-NO_3_ LDH and ZnAl-Cl_2_ LDH, and 48 h for MgAl–Cl_2_ LDH.

### 2.3. Bacteria Assay

Tests were conducted using *Escherichia coli* (NCTC 9001, isolated from human urine, cystitis), *Staphylococcus aureus* (NCTC 13811, isolated from human wound infection), and *Pseudomonas aeruginosa* (NCTC 14478, reference strain) strains, selected for their relevance in antimicrobial and biofilm research.

Overnight cultures in Nutrient Broth (Sigma-Aldrich, St. Louis, MO, USA) were adjusted to a concentration of 10^6^ CFU mL^−1^ using the 0.5 McFarland standard. For each bacteria type, 1 mL of overnight culture was added to a cuvette alongside 2 mL of DI water, producing a solution with a dilution factor of 3. The optical density of the cuvette is then measured with a cell density metre, against a reference of 3 mL DI water inside a different cuvette. The optical density (OD) obtained is then used to calculate the CFU mL^−1^ of the overnight solution as per the 0.5 McFarland standard. The CFU mL^−1^ obtained is then used to calculate the amount of volume to collect from the overnight solution to obtain a bacteria solution with a CFU mL^−1^ of 10^6^. This is the calibrated solution used to inoculate the samples.

Both etched-glass LDH samples and in situ LDH samples were cut to size to fit a 12-well culture plate and 3 mL of the calibrated bacterial suspension was added to each well.

Non LDH-treated glass coverslips were used as control samples. An additional set of control sample, consisting of etched-glass coverslip without LDH deposition (“etched blank” sample) was used to verify the absence of antimicrobial effect due to the etching process. No additional control (e.g., bare aluminium substrate) was included. In the in situ synthesis approach, the aluminium substrate does not act as a passive surface but is partially consumed during the LDH formation process, serving as a precursor for the growth of the MgAl- or ZnAl-based layered double hydroxide. As a result, the exposed surface is no longer representative of bare aluminium, but of the resulting LDH coating. Moreover, the absence of antimicrobial activity in pure aluminium has been reported in the literature [34]. Consequently, an untreated aluminium control would not provide a meaningful baseline for comparison with the in situ-grown samples.

Samples were incubated at 37 °C for 24 h to allow preliminary surface colonisation and early biofilm-like structure formation. The 24h timepoint has been chosen as it is an established timepoint for antimicrobial surface studies [35,36,37]. Following incubation, samples were gently washed with phosphate-buffered saline (PBS) to remove planktonic cells and transferred to a new well plate containing fresh Nutrient Broth.

Biofilms were detached from the sample surfaces by 15 min sonication at 45 kHz, following the biofilm detachment protocol described in [38]. The resulting bacterial suspensions were serially diluted and plated following the Miles and Misra method [39]. A total of 100 µL of sample bacteria solution is transferred into 900 µL of Nutrient Broth, generating six consecutive dilutions. For each dilution, three 10 µL aliquots were spotted onto Nutrient Agar (Sigma-Aldrich, St. Louis, MO, USA) plates. Plates were incubated at 37 °C for a further 24 h, after which colonies were counted using a digital colony counter. This protocol results in three colony counts per sample.

### 2.4. Statistical Analysis

To obtain statistically relevant results, the procedure described in Section 2.3 was independently repeated three times for each sample, corresponding to three biological replicates. For each biological replicate, three colony counts were obtained, giving a total of nine colony counts per sample.

The colony counts were used to calculate the average number of colony-forming units (CFU) per millilitre and the standard deviation of the colony counts obtained from the assay.

CFU data were Log_10_-transformed prior to analysis. Differences between controls and LDH formulations were assessed using one-way ANOVA followed by Tukey–Kramer post hoc testing. Analyses were performed separately for each bacteria strain and for each sample type (etched-glass and in situ-grown). Data are presented as mean ± 95% CI (*n* = 9 biological replicates).

### 2.5. Materials Characterisation

LDH samples were characterised using X-ray diffraction (XRD), scanning electron microscopy (SEM), energy-dispersive X-ray spectroscopy (EDS), and contact angle analysis.

XRD analysis was performed using a Rigaku SmartLab SE X-ray diffraction system. A total of θ–2θ scans were collected over the range 5–75° at a scan rate of 4° min^−1^ with a step size of 0.02°. Lattice parameters *a* and *c* were determined from the resulting diffraction patterns.

SEM imaging was carried out using both a JEOL JSM-7001F (Tokyo, Japan) field-emission scanning electron microscope and a TESCAN Vega (Brno, Czech Republic) scanning electron microscope. For etched-glass samples, a sacrificial slide was cut into 1 mm × 1 mm sections to fit onto SEM stubs, while 1 mm × 1 mm aluminium sections were prepared for in situ-synthesised samples. All samples were sputter-coated with a 10 nm chromium layer prior to imaging. EDS analysis (Xplore EDS detector, Oxford Instruments, Oxfordshire, UK) was conducted on the same samples at multiple locations to assess elemental composition, coating homogeneity, and the absence of contamination. EDS data was processed using the AZtecLive software (Oxford Instruments, Oxfordshire, UK).

### 2.6. Contact Angle

Wettability and hydrophobicity data of the samples were acquired with a contact angle goniometer (Attension, Biolin Scientific, Gothenburg, Sweden) using the sessile drop method. A controlled volume of water was deposited onto the surface through a precision syringe and the resulting profile of the droplet was captured with a high resolution camera over a 10 s period. This was made to assess any dynamic wetting behaviour present in the samples. The contact angle values were determined by fitting the right and left profile of the droplet to an appropriate tangent line by using the built-in software (OneAttension, Biolin Scientific, Gothenburg, Sweden), and the presented contact angle values represent the average between the angle formed by the right and left fitted lines with the surface. Three samples of each type were analysed, and their mean values were averaged to obtain a statistically relevant measure of their wettability.

### 2.7. Bacterial Adhesion and Biofilm Formation Imaging

Bacterial adhesion and biofilm formation on LDH samples was observed using scanning electron microscopy (SEM). SEM is commonly regarded as a gold-standard technique in antimicrobial and biofilm research, as it enables high-resolution visualisation of bacterial morphology, surface attachment, and biofilm architecture, providing direct evidence of bacteria–material interactions that cannot be resolved by bulk or optical assays alone.

Overnight cultures of *Escherichia coli* (NCTC 9001, isolated from human urine, cystitis), *Staphylococcus aureus* (NCTC 13811, isolated from human wound infection), and *Pseudomonas aeruginosa* (NCTC 14478, reference strain) were calibrated to a concentration of 10^6^ CFU mL^−1^ using the 0.5 McFarland standard, following the same procedure described in 2.3. LDH-treated etched-glass samples and in situ-grown samples were cut to size to fit into a 12-well culture plate and inoculated with 1 mL of the calibrated bacterial suspension per well. Samples were incubated at 37 °C for 24 h.

Following incubation, the bacterial suspension was removed, and samples were fixed using a 2.5% glutaraldehyde solution in phosphate-buffered saline (PBS), ensuring complete coverage of each sample. The plate was then stored at 4 °C overnight. The fixative was subsequently discarded and samples were washed three times with PBS.

Dehydration was performed via a graded ethanol series in deionised water (50%, 70%, and 95%), with each dilution applied twice for 10 min under agitation. This was followed by three washes in 100% ethanol, each lasting 15 min under agitation. Chemical dehydration was then completed using hexamethyldisilane (HMDS), applied sequentially as ethanol:HMDS mixtures (2:1, 1:1, and 1:2, *v*/*v*), each for 15 min, followed by three final washes in absolute HMDS for 15 min each. After removal of HMDS, samples were allowed to air dry overnight.

Dried samples were mounted onto SEM stubs using carbon tape and sputter-coated with a 10 nm gold layer prior to SEM imaging.

## 3. Results

### 3.1. XRD Characterisation

**Figure 3:**
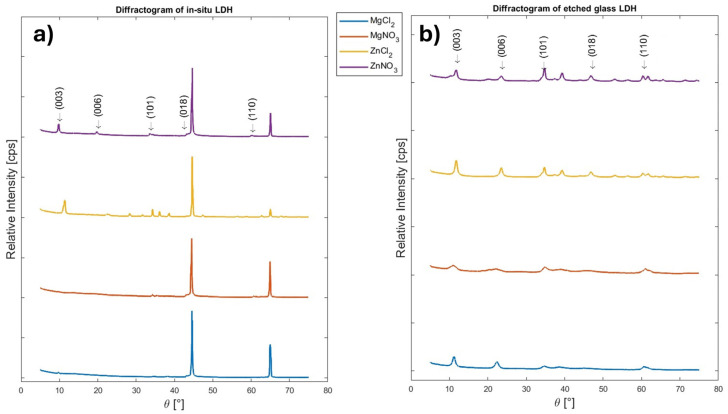
Diffractograms; (**a**) in situ LDH; (**b**) etched-glass LDH.

Figure 3 shows the XRD diffractograms of all the four formulations of LDH-synthesised both with in situ-grown and coprecipitation methods. It is possible to notice that all formulations tested exhibit XRD peaks consistent with LDH structure. The *a* lattice parameter recorded ranges between 3.02 Å and 3.07 Å for all samples, while *c* values are approximately 23.6 Å for etched samples and 27.5 Å for in situ-grown samples. These values are consistent with those reported in the literature for MgAl- and ZnAl-based LDH systems [29,31].

The limited variation in terms of the lattice parameter *a* observed among the different LDH formulations for both synthesis methods indicates the formation of well-defined crystalline LDH structures across all samples.

The values of *c* represent three times the basal spacing, in the so-called 3R polytype stacking, where subsequent *ab* planes are rotated 120° between each other, resulting in an A-B-C-A… sequence. Differences in the *c* lattice parameter between etched and in situ samples can be attributed to the distinct synthesis processes. In situ synthesis provides a continuous aluminium source and a large reactive surface, promoting LDH nucleation and growth directly at the substrate interface. In contrast, LDH coprecipitation, subsequent deposition on etched glass followed by annealing potentially influence layer stacking and interlayer spacing, resulting in a higher *c* value.

### 3.2. SEM Characterisation

**Figure 4:**
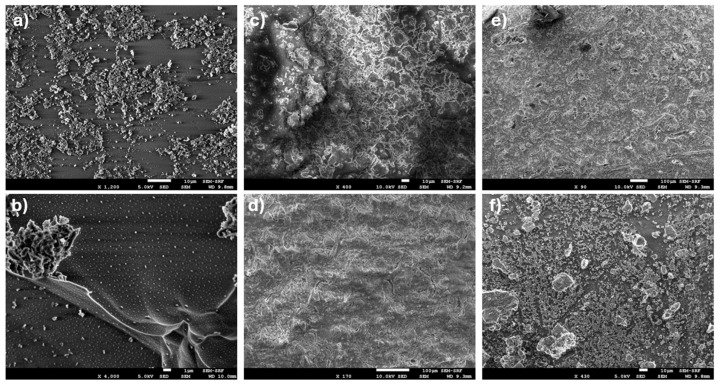
SEM images showing surface topography of all LDH-treated etched-glass samples; (**a**) etched-glass without LDH deposition and (**b**) detail of structure produced from hydrofluoric acid etching, showing rough surface texture and sudden height step; (**c**) MgAl-NO_3_ LDH; (**d**) MgAl-Cl_2_ LDH; (**e**) ZnAl-NO_3_ LDH; and (**f**) ZnAl-Cl_2_ LDH.

**Figure 5:**
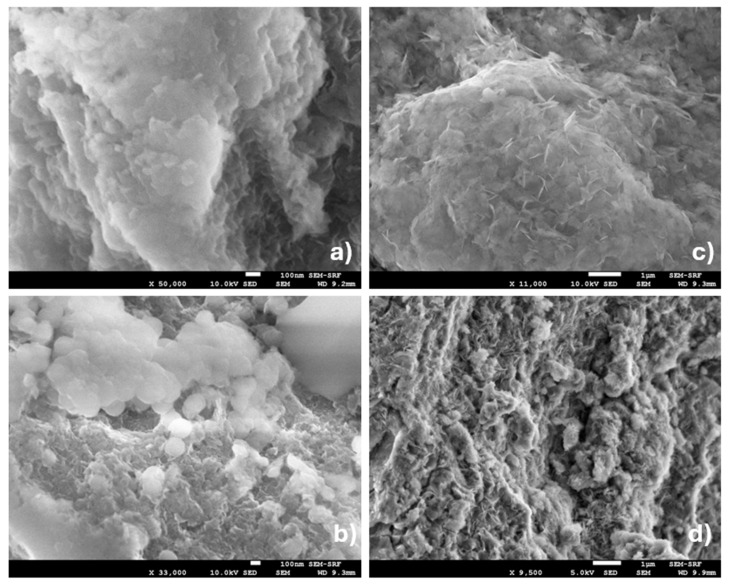
SEM images of LDH-treated etched glass at higher magnification exhibiting the leaf-like nanostructure; (**a**) MgAl-NO_3_ LDH; (**b**) MgAl-Cl_2_ LDH; (**c**) ZnAl-NO_3_ LDH; and (**d**) ZnAl-Cl_2_ LDH.

SEM analysis revealed that all etched LDH samples were uniformly covered by LDH coatings (Figure 4). At higher magnification (Figure 5), LDH-treated samples exhibited the characteristic lamellar, platelet-like morphology typical of layered double hydroxides, distributed across the etched substrate. In contrast, non-treated etched-glass (“etched blank”) samples displayed the effects of hydrofluoric acid etching alone, characterised by a porous surface with larger crystalline features, a rough underlying layer, and occasional abrupt height variations. The morphology reflects the influence of substrate micro- and nano-scale roughness introduced during the etching process. While the LDH retains its intrinsic leaf-like lamellar structure, platelet attachment and aggregation are spatially heterogeneous and follow the underlying surface relief, resulting in coatings with pronounced local height variations and uneven coverage.

**Figure 6:**
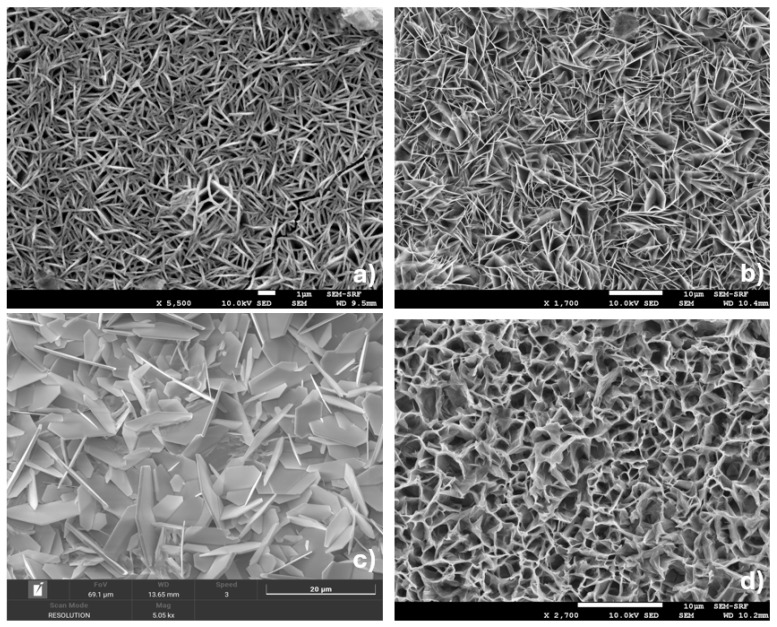
SEM images of in situ synthesis LDH; (**a**) MgAl-Cl_2_; (**b**) MgAl-NO_3_; (**c**) ZnAl-Cl_2_; (**d**) ZnAl-NO_3_.

In situ-grown LDH samples (Figure 6) also showed continuous nanomaterial coverage across the substrate. The coatings consisted of uniformly distributed nanoplatelets with consistent size and coverage. The morphology appears to be primarily governed by growth conditions rather than substrate topography. Nucleation density and lamellar development are influenced by parameters such as reaction time, temperature, precursor concentration, and substrate orientation, leading to more homogeneous coatings with reduced apparent height variation under the conditions employed in this study. Distinct differences in the leaf-like structures are observed between formulations. ZnAl–LDH samples differ from MgAl–LDH in both platelet size and morphology. ZnAl–NO_3_ platelets exhibit jagged edges and pointed features, which may influence bacterial adhesion and interaction with the surface. ZnAl–Cl_2_ presents larger and more planar platelets compared to other formulations, providing a more extended surface for potential bacterial attachment. MgAl–LDH samples display sharp-edged platelets of comparable morphology, with MgAl–Cl_2_ showing smaller platelet dimensions relative to MgAl–NO_3_.

### 3.3. EDS Characterisation

**Figure 7:**
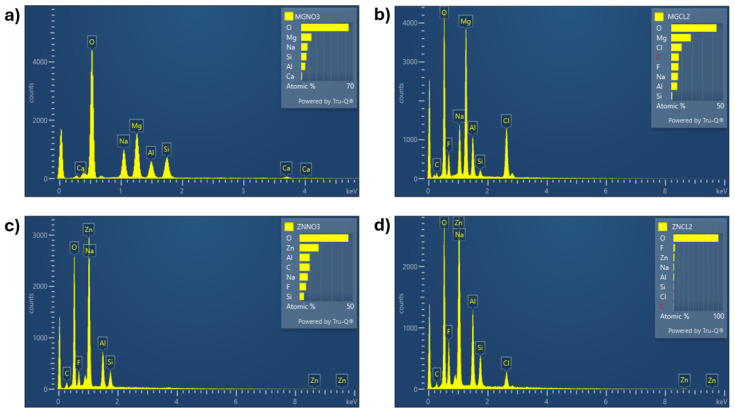
EDS spectra of etched LDH samples: (**a**) MgAl-NO_3_; (**b**) MgAl-Cl_2_; (**c**) ZnAl-NO_3_; and (**d**) ZnAl-Cl_2_.

Energy-dispersive X-ray spectroscopy (EDS) confirmed the expected elemental composition of the etched-glass LDH coatings (Figure 7). The divalent metal cation corresponding to each formulation, magnesium in MgAl-LDH and zinc in ZnAl-LDH, was also detected. In chloride-intercalated LDHs (MgAl–Cl_2_ and ZnAl–Cl_2_; Figure 7b and Figure 7d, respectively), a distinct chlorine peak was observed, confirming successful chloride intercalation. In contrast, nitrogen peaks were not detected in the nitrate-intercalated samples (MgAl–NO_3_ and ZnAl–NO_3_; Figure 7a,c).

Additional elements observed in the spectra can be attributed to synthesis and substrate preparation. Sodium likely originates from residual sodium hydroxide used during coprecipitation, while fluorine arises from hydrofluoric acid employed during glass etching. The presence of silicon (and calcium) reflects contributions from the underlying glass substrate, which is expected due to the finite interaction volume of EDS and the thin, lamellar nature of the LDH coatings, particularly on etched surfaces.

**Figure 8:**
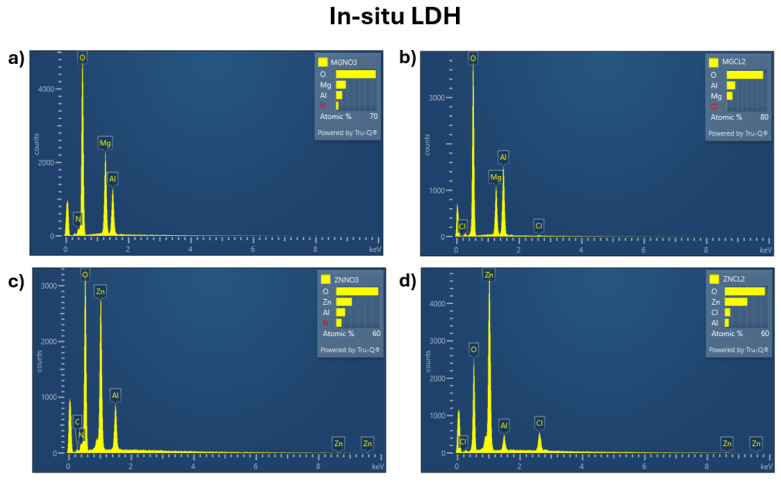
EDS spectra of in situ LDH; (**a**) MgAl-NO_3_; (**b**) MgAl-Cl_2_; (**c**) ZnAl-NO_3_; and (**d**) ZnAl-Cl_2._

Representative EDS spectra for the in situ LDH samples are shown in Figure 8. The results for the in situ LDH samples confirm the presence of elements consistent with the specific formulation tested and are comparable with those exhibited by etched-glass samples but lacked detectable calcium and fluorine. The absence of calcium and fluorine reflects the different preparation route, as these coatings were grown directly on aluminium substrates without hydrofluoric acid etching and therefore contain no residues from glass or etching reagents.

### 3.4. Contact Angle Analysis

A contact angle analysis was performed on all LDH samples to assess surface wettability, a factor known to influence bacterial adhesion and the onset of biofilm formation.

**Figure 9:**
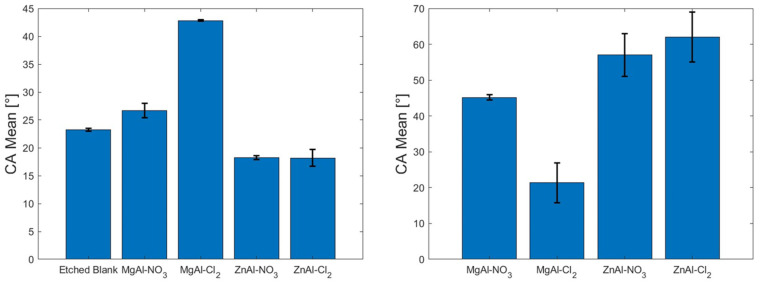
Mean values of contact angle for etched-glass (**left**) and in situ (**right**) LDH samples with standard deviation bars.

As per contact angle analysis, a mean value greater than 90° indicates hydrophobicity of the surface and lesser wettability, whereas an angle lesser than 90° indicates hydrophilicity and greater wettability [40]. All LDH coatings, whether deposited on etched glass or formed in situ, exhibited mean static contact angles below 90° (Figure 9). Among the etched-glass samples, MgAl–Cl_2_ displayed the highest mean contact angle, approximately 43°, indicating a relatively less hydrophilic behaviour when compared with the other etched-glass coatings. Conversely, this same formulation showed the lowest mean contact angle among the in situ-grown samples. Apart from MgAl–Cl_2_, all in situ coatings presented higher mean contact angles than their etched-glass counterparts.

**Figure 10:**
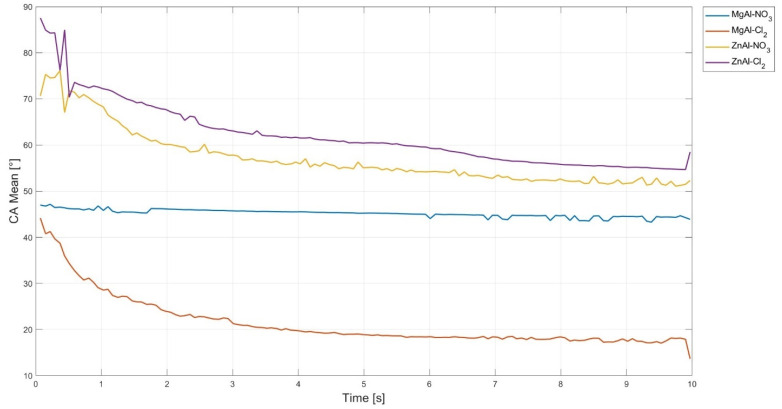
Mean contact angle for in situ samples.

**Figure 11:**
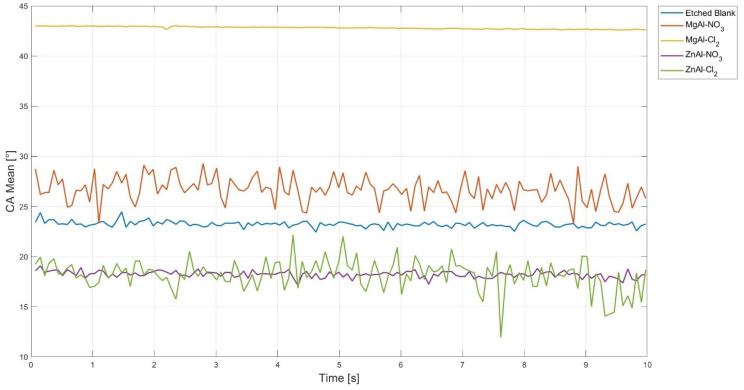
Mean contact angle for etched-glass samples.

Mean values reported for the in situ samples reflect a markedly transient wetting behaviour (Figure 10). To account for this initial transient response, mean contact angles were calculated from 4 s onwards, after which the measurements stabilised. The adjusted contact angle data are summarised in Table 1. In contrast, the etched-glass samples exhibited stable contact angles over time (Figure 11), and no correction was applied to their mean values.

### 3.5. Bacteria Assays

#### 3.5.1. Etched LDH Samples

**Figure 12:**
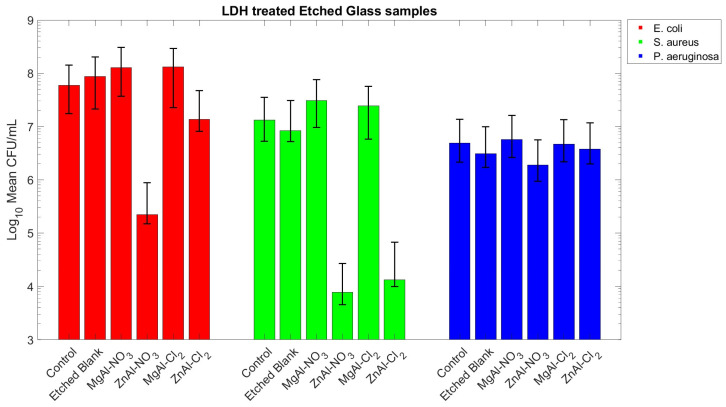
Mean CFU mL^−1^ (Log_10_ scale) for each LDH-treated etched-glass sample, grouped by bacterial strain. Error bars represent the standard deviation of three biological replicates per sample (*n* = 9 colony counts).

Figure 12 shows the results of the bacterial biofilm assays performed on LDH-treated etched-glass substrates. The results indicate that surface etching alone does not significantly affect biofilm formation, as CFU mL^−1^ values obtained for etched blank samples are comparable to those of the control across all bacterial strains tested.

Amongst the LDH formulations tested, ZnAl-based samples exhibited the highest antimicrobial activity, consistent with the well-documented antimicrobial properties of zinc. Nitrate-intercalated ZnAl-LDH induced an approximately three-log reduction in CFU mL^−1^ for both *E. coli* and *S. aureus*. Similarly, chloride-intercalated ZnAl-LDH produced a three-log reduction in *S. aureus* biofilm formation, while its effectiveness against *E. coli* was less pronounced. In contrast, both nitrate- and chloride-intercalated MgAl-LDHs did not inhibit biofilm formation; CFU mL^−1^ values were comparable to, or higher than, those of the control samples, indicating no antimicrobial effect under the conditions tested.

The antimicrobial behaviour of etched LDH coatings is primarily governed by their chemical composition, with ZnAl-based formulations consistently outperforming MgAl counterparts against *E. coli* and *S. aureus*. This trend is consistent with the known antimicrobial activity of zinc-containing materials, which can disrupt bacterial membranes, interfere with metabolic pathways, and inhibit biofilm development. In contrast, MgAl-based LDHs do not provide a comparable inhibitory environment, resulting in higher bacterial viability and more extensive biofilm formation under the same conditions. Across all LDH formulations, no statistically significant reduction in *P. aeruginosa* biofilm formation was observed, with CFU mL^−1^ values remaining comparable to controls. This behaviour is consistent with the intrinsic resistance of this strain, which is associated with its strong biofilm-forming capability and the production of extracellular polymeric substances (EPSs) that facilitate surface colonisation even under adverse conditions. The limited sensitivity of *P. aeruginosa* suggests that the antimicrobial mechanisms active in these LDH systems are insufficient to overcome its inherent defence strategies.

#### 3.5.2. Statistical Analysis of Etched LDH Samples Results

One-way ANOVA of CFU counts revealed significant differences between groups for *E. coli* and *S. aureus* (F_Ec_ (5, 48) = 40.2, p_Ec_ = 4.8 × 10^−16^, $\eta_{Ec}^{2}$ = 0.81; F_Sa_(5, 48) = 22.8, p_Sa_ = 6.1 × 10^−12^, $\eta_{Sa}^{2}$ = 0.71), whereas no statistical differences between groups have been observed for *P. aeruginosa* (F_Pa_ (5, 48) = 4.7, p_Pa_ = 0.06, $\eta_{Pa}^{2}$ = 0.19). Because zero values are undefined under log_10_ transformation, zero counts (e.g., complete kill) were replaced with a small positive pseudocount corresponding to a single count in the lowest dilution (1 × 10^1^ CFU mL^−1^) prior to transformation. These zero counts occurred for ZnAl-NO_3_ samples in *E. coli* and ZnAl-Cl_2_ samples in *S. aureus*.

Post hoc Tukey–Kramer comparisons for *E. coli* showed that ZnAl-NO_3_ was significantly lower than control (mean difference = −4.7, 95% CI [−6.0, −3.4]), etched blank sample (mean difference = −4.8, 95% CI [−6.2, −3.6]), MgAl-NO_3_ (mean difference = −5.0, 95% CI [−6.3, −3.7]), ZnAl-Cl_2_ (mean difference = −3.9, 95% CI [−5.2, −2.6]), and MgAl-Cl_2_ (mean difference = −5.1, 95% CI [−6.4, −3.8]). No other pairwise differences reached significance.

For *S. aureus*, ZnAl-Cl_2_ was significantly lower than control (mean difference = −4.5, 95% CI [−6.3, −2.6]), etched blank (mean difference = −2.8, 95% CI [−4.6, −0.9]), MgAl-NO_3_ (mean difference = −4.8, 95% CI [−6.7, −3.0]), and MgAl-Cl_2_ (mean difference = −4.8, 95% CI [−6.6, −2.9]). The etched blank sample was significantly lower than MgAl-NO_3_ (mean difference = −2.1, 95% CI [−3.9, −0.2]), and MgAl-Cl_2_ (mean difference = −2.0, 95% CI [−3.8, −0.2]), but significantly higher than ZnAl-NO_3_ (mean difference = 1.9, 95% CI [0.1, 3.8]). Additionally, ZnAl-NO_3_ was significantly lower than MgAl-NO_3_ (mean difference = −4.1, 95% CI [−5.9, −2.2]), and MgAl-Cl_2_ (mean difference = −4.0, 95% CI [−5.8, −2.2]). No other pairwise differences reached significance.

For *P. aeruginosa*, no significant pairwise differences were observed.

#### 3.5.3. In Situ LDH Samples

**Figure 13:**
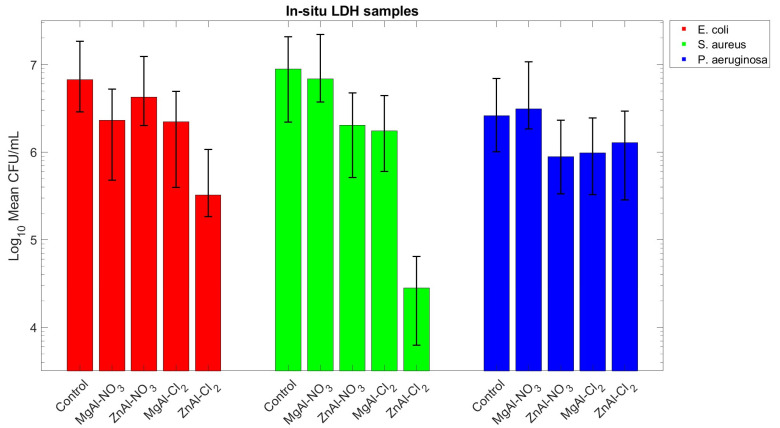
Mean CFU mL^−1^ (Log_10_ scale) for each in situ LDH formulation, grouped by bacterial strain. Error bars represent the standard deviation of three biological replicates per sample (*n* = 9 colony counts).

The results obtained for the in situ-grown LDH samples (Figure 13) show a similar trend to that observed for the etched-glass coatings against *S. aureus*, while also highlighting differences in overall antimicrobial performance. Zn-based in situ coatings exhibited pronounced reductions in viable counts, with ZnAl–Cl_2_ inducing an almost three-log reduction.

A different pattern emerged for *E. coli*. On etched-glass substrates, the strongest reduction in CFU mL^−1^ was observed for ZnAl–NO_3_ (approximately three log units), while ZnAl–Cl_2_ exhibited a weaker effect (approximately one log unit). In contrast, this trend was reversed for in situ-grown coatings, where ZnAl–Cl_2_ produced the largest decrease (around two log units) and ZnAl–NO_3_ resulted in a more modest, approximately one-log reduction relative to the control.

For *P. aeruginosa*, CFU mL^−1^ values remained comparable to those of the controls across all etched-glass and in situ formulations, indicating no significant inhibitory effect of either coating type against this strain.

#### 3.5.4. Statistical Analysis of In Situ LDH Samples Results

One-way ANOVA of CFU counts revealed significant differences between groups for *E. coli*, *S. aureus*, and *P. aeruginosa* (F_Ec_ (4, 40) = 17.9, p_Ec_ = 1.7 × 10^−8^, $\eta_{Ec}^{2}$ = 0.64; F_Sa_ (4, 40) = 78.1, p_Sa_ = 2.4 × 10^−18^, $\eta_{Sa}^{2}$ = 0.88; F_Pa_ (4, 40) = 3.6, p_Pa_ = 0.01, $\eta_{Pa}^{2}$ = 0.26).

Post hoc Tukey–Kramer comparisons for *E. coli* showed that ZnAl-Cl_2_ was significantly lower than control (mean difference = −1.8, 95% CI [−2.5, −1.1]), MgAl-NO_3_ (mean difference = −5.0, 95% CI [−6.3, −3.7]), ZnAl-NO_3_ (mean difference = −1.6, 95% CI [−2.3, −0.9]), and MgAl-Cl_2_ (mean difference = −1.4, 95% CI [−2.1, −0.7]). No other pairwise differences reached significance.

For *S. aureus*, control was significantly higher than ZnAl-NO_3_ (mean difference = 0.6, 95% CI [0.2, 1.1]), MgAl-Cl_2_ (mean difference = 0.7, 95% CI [0.3, 1.2]), and ZnAl-Cl_2_ (mean difference = 2.5, 95% CI [2.0, 2.9]). Comparisons also showed that ZnAl-Cl_2_ was significantly lower than MgAl-NO_3_ (mean difference = −2.1, 95% CI [−2.5, −1.6]), MgAl-Cl_2_ (mean difference = −1.7, 95% CI [−2.2, −1.3]), and ZnAl-NO_3_ (mean difference = −1.8, 95% CI [−2.3, −1.4]). No other pairwise differences reached significance.

For *P. aeruginosa*, control was significantly higher than ZnAl-NO_3_ (mean difference = 0.4, 95% CI [0.1, 0.9]). No other pairwise differences reached significance.

### 3.6. SEM Analysis

SEM observations of bacteria fixed on LDH were used as a qualitative assessment of bacteria colonisation and distribution across the surface of the samples. Although the SEM images confirm the trends observed for the bacterial assays performed, a quantitative analysis was not conducted, and therefore subsequent investigation should focus on quantifying surface coverage, roughness and bacterial occupancy to provide additional insight of the effect of the exposure of bacteria to LDH-treated surfaces.

#### 3.6.1. *E. coli*

**Figure 14:**
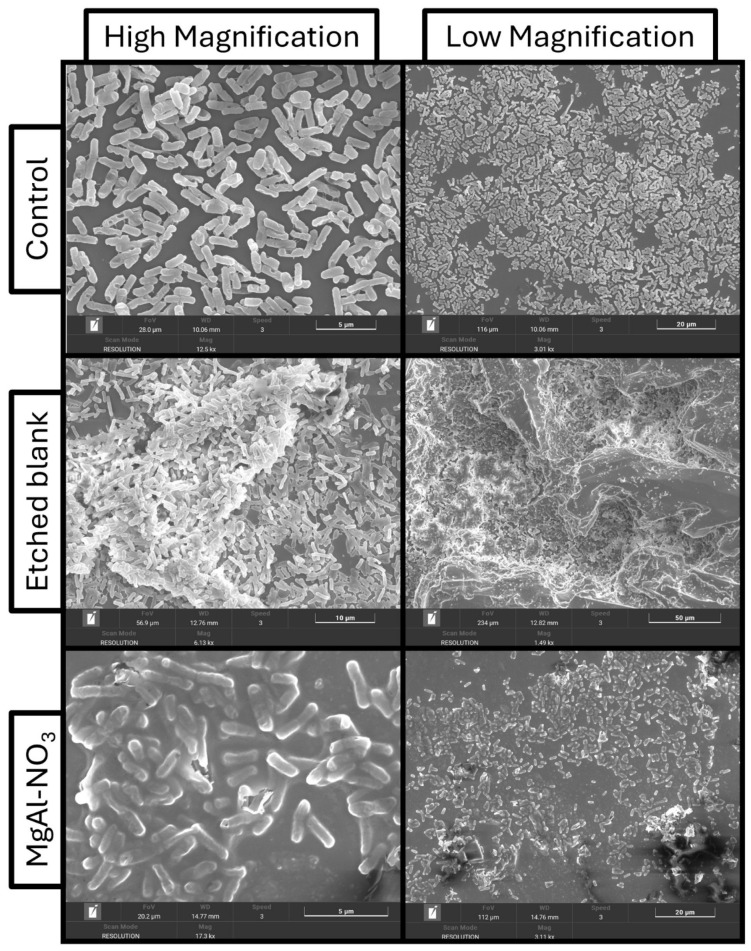
SEM images of *E. coli* etched-glass samples (control, etched blank and MgAl-NO_3_), both at high magnification (**left column**) and low magnification (**right column**).

**Figure 15:**
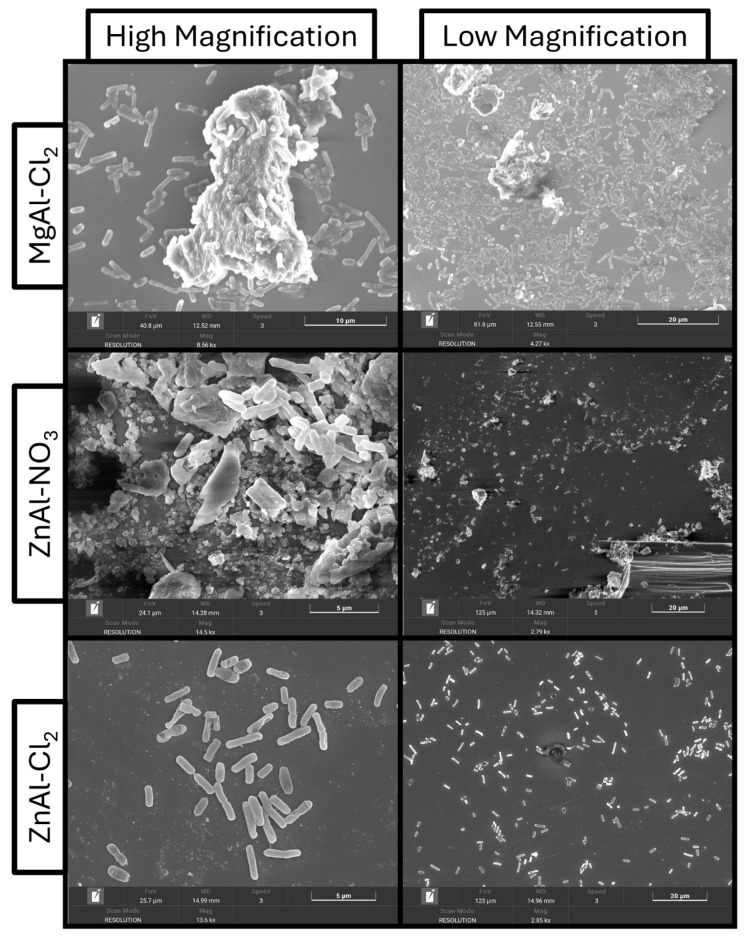
SEM images of *E. coli* etched-glass samples (MgAl-Cl_2_, ZnAl-NO_3_, ZnAl-Cl_2_), both at high magnification (**left column**) and low magnification (**right column**).

Representative SEM images of *E. coli* on the etched-glass samples are shown in Figure 14 and Figure 15. Occasional cells displaying membrane deformation were observed under all conditions and are likely artefacts of the fixation procedure rather than genuine structural damage. Both the untreated control and the etched-blank samples exhibited comparable distributions of well-separated bacterial cells, confirming that the etching process alone does not significantly influence *E. coli* colonisation or morphology. Bacterial clusters appeared to be preferentially localised near residual glass crystals generated during etching, a pattern also exhibited by the magnesium-based LDH coatings, where cells tended to accumulate on the regions of the substrate bearing MgAl-LDH. By contrast, the ZnAl-LDH coatings showed a marked reduction in bacterial colonisation. This effect was most pronounced on the ZnAl-NO_3_ sample, which displayed few adherent cells, predominantly isolated bacteria rather than aggregates. Furthermore, *E. coli* colonisation is reduced on ZnAl-based formulations compared to MgAl-based and control surfaces. ZnAl coatings are characterised by smaller and more dispersed bacterial aggregates, whereas MgAl-based samples exhibit more extensive clustering and surface coverage. On the ZnAl-Cl_2_ sample, bacteria were largely confined to less LDH-covered areas of the etched-glass surface, suggesting the effectiveness of LDH on reducing bacteria colonisation.

**Figure 16:**
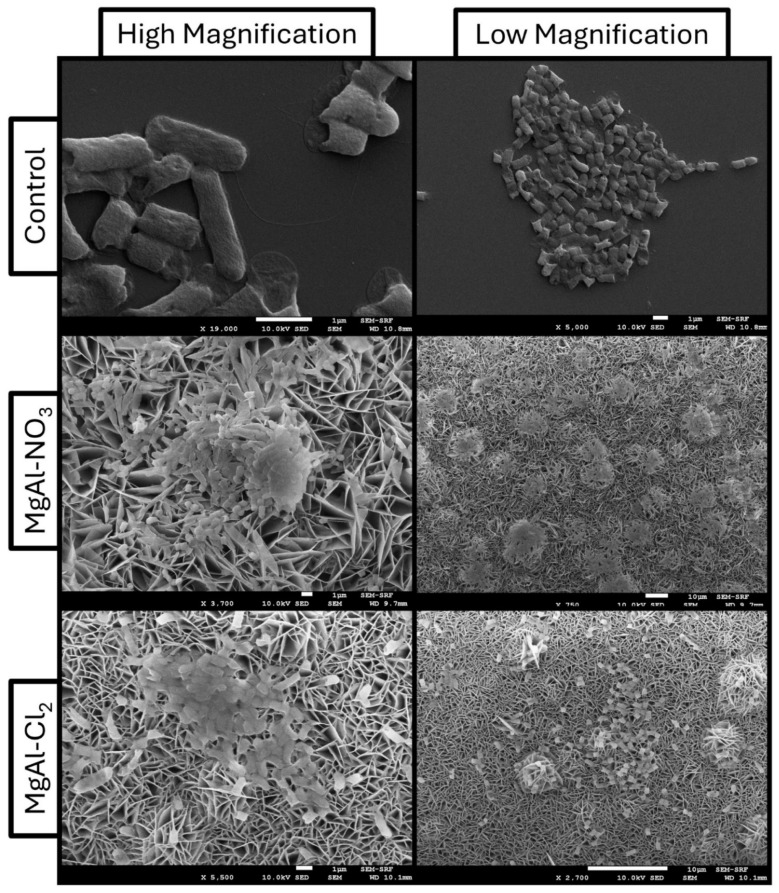
SEM images of *E. coli* in situ samples (control, MgAl-NO_3_, MgAl-Cl_2_), both at high magnification (**left column**) and low magnification (**right column**).

**Figure 17:**
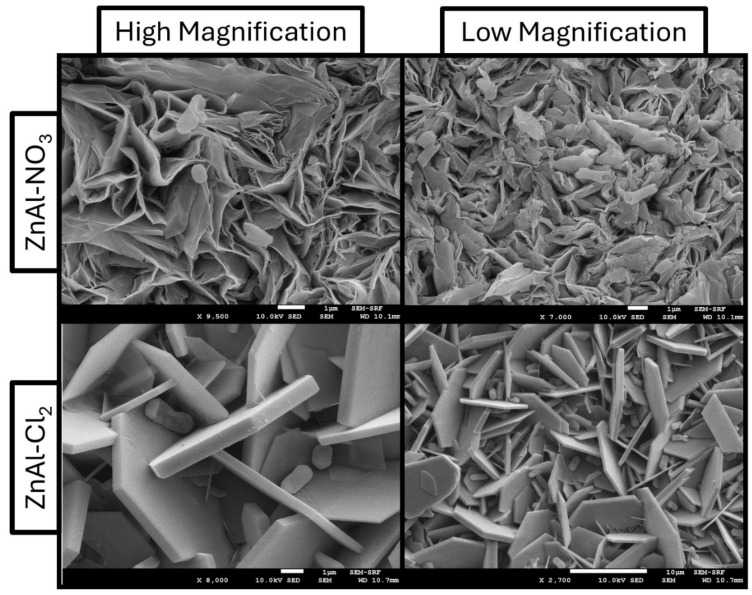
SEM images of *E. coli* in situ samples (ZnAl-NO_3_, ZnAl-Cl_2_), both in high magnification and low magnification.

SEM imaging analysis of *E. coli* exposed to in situ LDH samples is shown in Figure 16 and Figure 17. The distribution of bacteria on the control coverslip follows a similar trend to that observed for the etched-glass control, exhibiting well-defined aggregates of bacterial cells and early-stage biofilm formation. A comparable pattern is observed for the MgAl–LDH samples, where bacteria proliferate across the entire surface.

In contrast, ZnAl–LDH samples display a distinct behaviour. The morphology of the petal-like structures appears to hinder bacterial proliferation, particularly for ZnAl–Cl_2_, where *E. coli* is predominantly located between the leaf-like features rather than on top of them, as observed in the other samples.

#### 3.6.2. *S. aureus*

**Figure 18:**
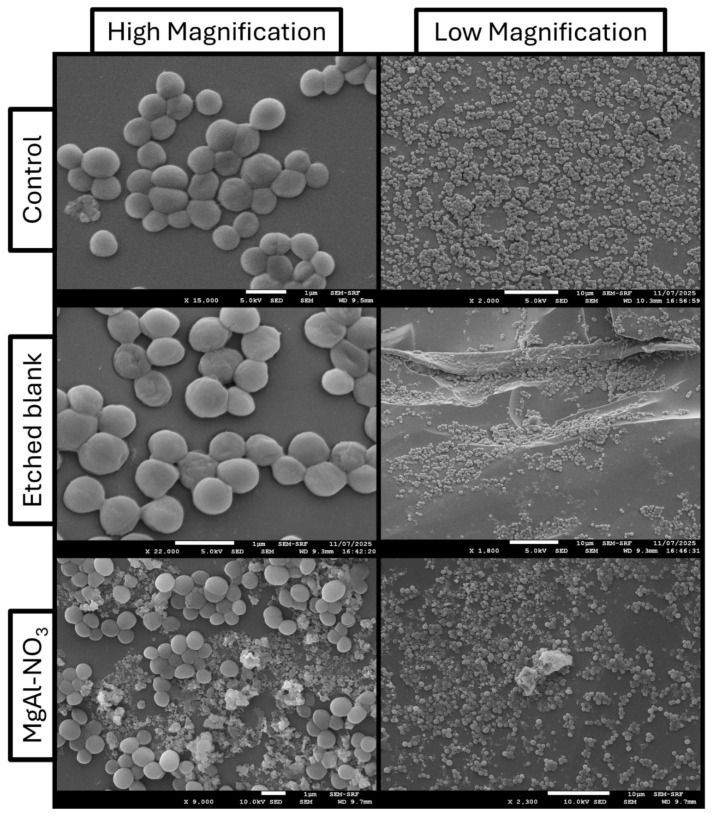
SEM images of *S. aureus* etched-glass samples (control, etched blank, MgAl-NO_3_), both at high magnification (**left column**) and low magnification (**right column**).

**Figure 19:**
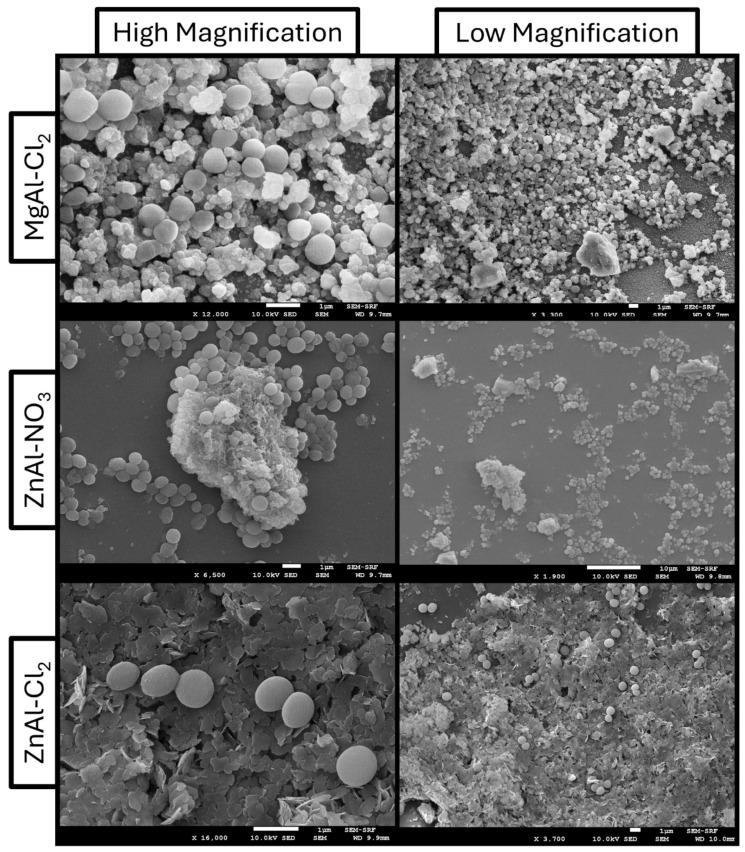
SEM images of *S. aureus* etched-glass samples (MgAl-Cl_2_, ZnAl-NO_3_, ZnAl-Cl_2_), both at high magnification (**left column**) and low magnification (**right column**).

Figure 18 and Figure 19 present SEM images of *S. aureus* colonisation on the etched-glass samples, showing a marked reduction in *S. aureus* coverage on ZnAl-based samples compared to MgAl formulations. As with the previous strain, the etched-blank samples displayed colony densities comparable to the untreated controls, confirming that the etching process alone does not impart measurable antimicrobial activity. On ZnAl coatings, bacteria are predominantly observed as isolated cells or small aggregates, whereas MgAl-based surfaces support more continuous coverage, often associated with the exposed lamellar structures. This behaviour is in agreement with the quantitative assay results, while also exhibiting larger and more numerous aggregates of bacteria on magnesium-based samples.

**Figure 20:**
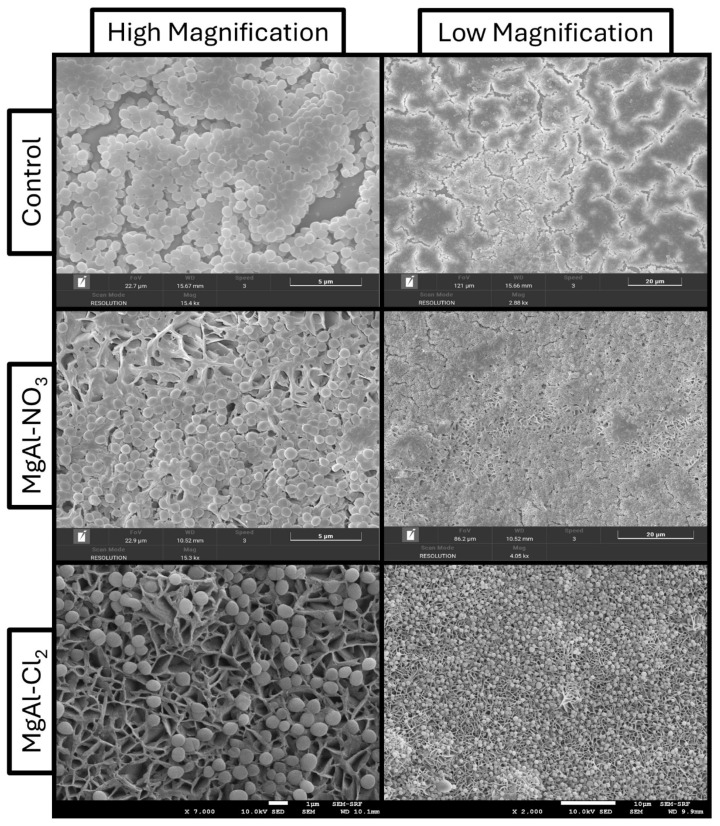
SEM images of *S. aureus* in situ samples (control, MgAl-NO_3_, MgAl-Cl_2_), both in high magnification and low magnification.

**Figure 21:**
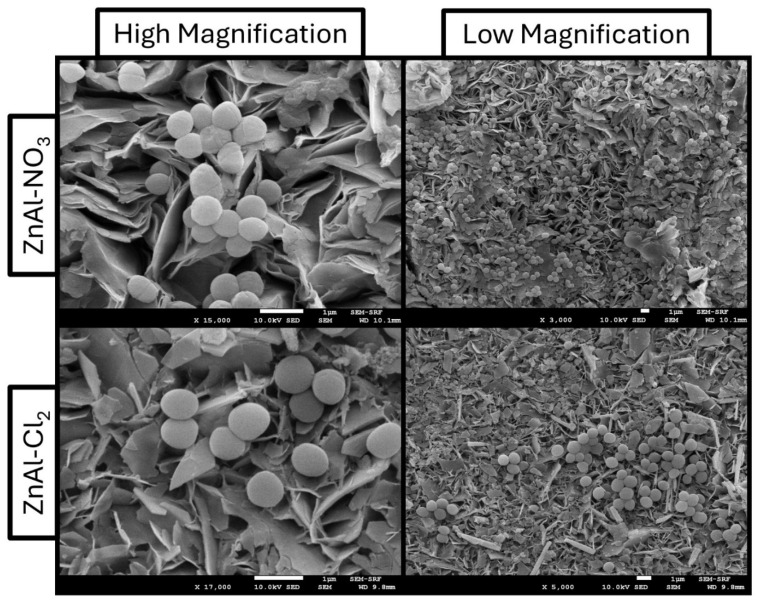
SEM images of *S. aureus* in situ samples (ZnAl-NO_3_, ZnAl-Cl_2_), both in high magnification and low magnification.

Figure 20 and Figure 21 show SEM images for *S. aureus* in situ LDH samples. The control coverslip shows sparse aggregates of bacteria with biofilm formation confirmed in various sites of the surface. A similar trend can be observed in the MgAl-LDH samples, with higher presence of bacteria on the surface of MgAl-Cl_2_ LDH. On the other hand, the ZnAl-LDH show a much lower concentration of cells throughout the surface, with occasional early-stage biofilm structures present only in confined areas of the sample. ZnAl–Cl_2_, in particular, shows sparse bacterial distribution across the surface, while MgAl-based coatings display more widespread colonisation. Differences in bacterial localisation are also evident, with ZnAl surfaces promoting confinement within interlamellar regions and MgAl surfaces favouring accumulation on top of platelets.

#### 3.6.3. *P. aeruginosa*

**Figure 22:**
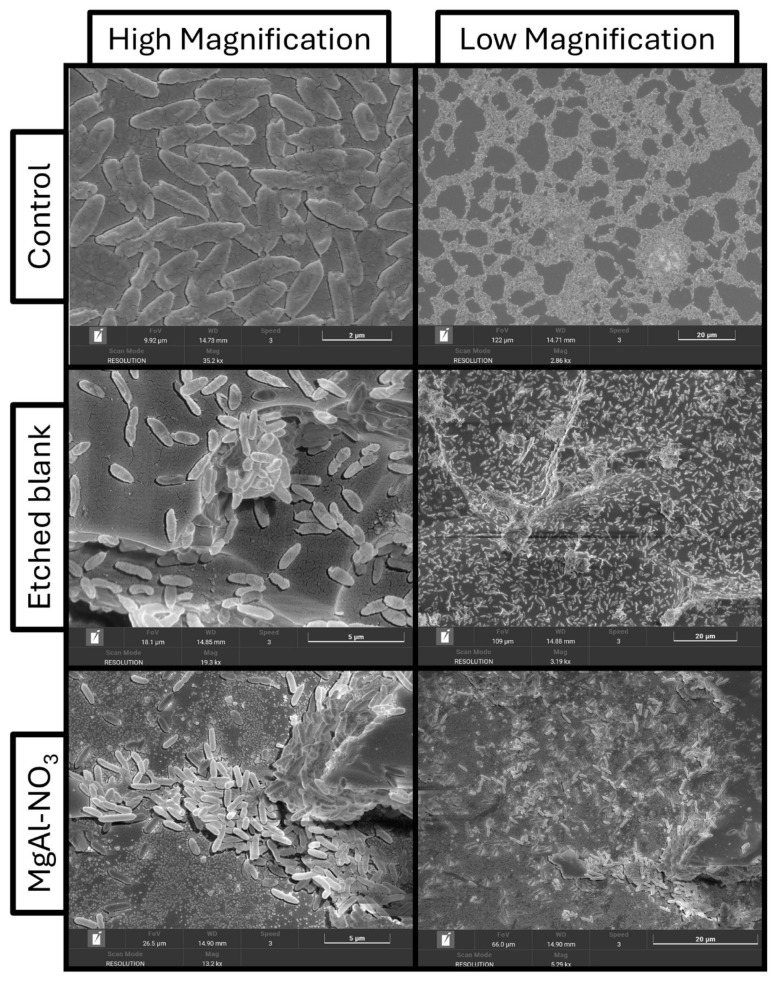
SEM images of *P. aeruginosa* etched-glass samples (control, etched blank, MgAl-NO_3_), both at high magnification (**left column**) and low magnification (**right column**).

**Figure 23:**
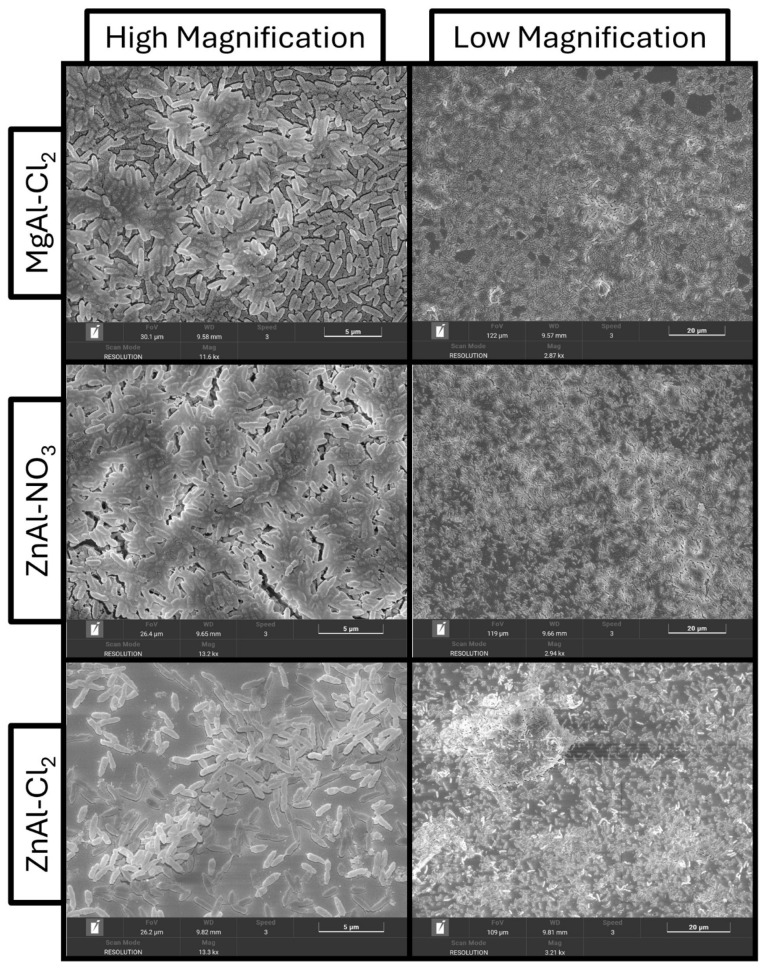
SEM images of *P. aeruginosa* etched-glass samples (MgAl-Cl_2_, ZnAl-NO_3_, ZnAl-Cl_2_), both at high magnification (**left column**) and low magnification (**right column**).

Figure 22 and Figure 23 show *P. aeruginosa* colonisation on etched LDH samples. The control and etched blank images show substantial bacterial proliferation, with numerous cell aggregates and biofilm structures distributed across the surface. In contrast, etched LDH samples exhibit sparser but still clearly present bacterial aggregates, with little variation between formulations, confirming the absence of a significant antimicrobial effect against *P. aeruginosa*.

**Figure 24:**
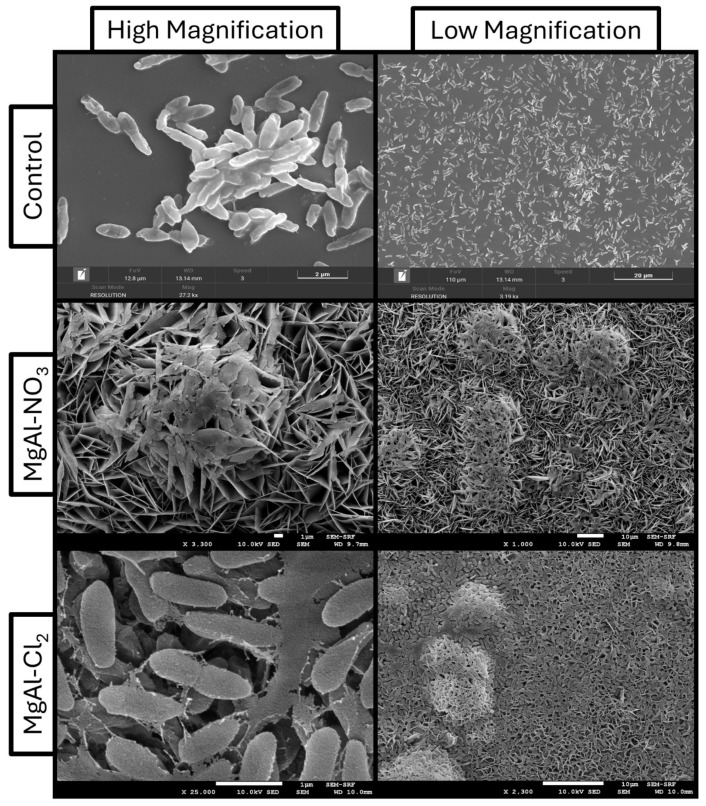
SEM images of *P. aeruginosa* in situ samples (control, MgAl-NO_3_, MgAl-Cl_2_), both at high magnification (**left column**) and low magnification (**right column**).

**Figure 25:**
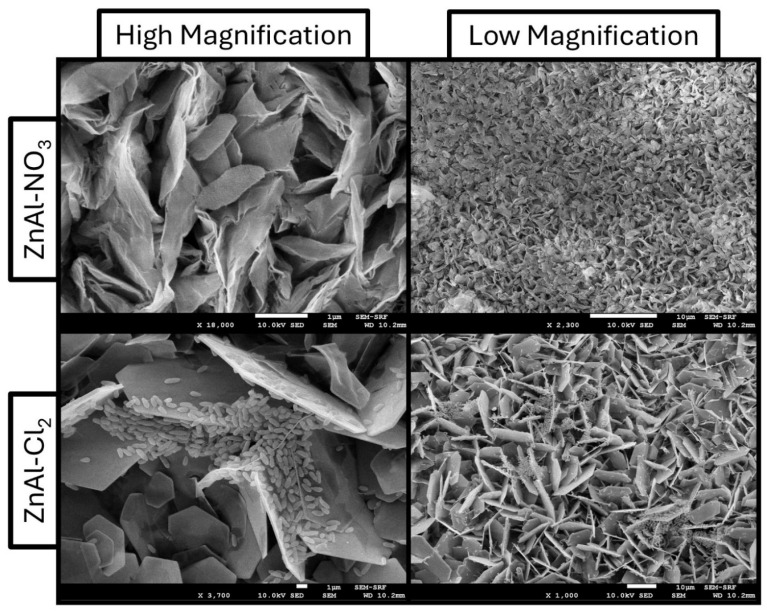
SEM images of *P. aeruginosa* in situ samples (ZnAl-NO_3_, ZnAl-Cl_2_), both at high magnification (**left column**) and low magnification (**right column**).

Figure 24 and Figure 25 show SEM images of *P. aeruginosa* on in situ LDH samples, confirming the trend observed for etched LDH surfaces. In MgAl–LDH samples, bacterial proliferation is either localised around LDH nucleation sites or distributed across the entire surface. In contrast, ZnAl–LDH samples show a more confined distribution, with bacteria predominantly located within the lamellar structures rather than on top of them. While variations in surface morphology and wettability influence local bacterial organisation, these effects do not translate into a measurable reduction in overall colonisation.

## 4. Discussion

The characterisation results obtained in this study are in good agreement with previous reports on Al-based LDH materials, supporting the successful formation of the intended LDH structures.

SEM analysis revealed distinct surface topographies across the tested samples, reflecting differences in both LDH formulation and synthesis route.

Although differences in platelet size and morphology are likely related to variations in chemical composition, differences in synthesis conditions, particularly reaction time, may also contribute. Variations in growth kinetics between formulations could influence platelet development, with longer synthesis times potentially leading to larger structures [29,31], as suggested by the observed differences between ZnAl–Cl_2_ and MgAl–Cl_2_.

Chemical composition of the synthesised products is confirmed by both EDS and XRD, showing expected elemental and crystalline peaks for all LDH formulations. For ZnAl–NO_3_ LDH, the absence of a detectable nitrogen signal in both in situ and etched-glass samples can be attributed to the limited sensitivity of EDS to light elements such as nitrogen, which produce weak X-ray signals that are easily masked. In addition, partial anion exchange with atmospheric carbonate or other species during synthesis and post-processing may reduce the near-surface nitrate concentration. Given that EDS primarily probes the near-surface region, nitrate located deeper within the interlayer structure may remain undetected. Nevertheless, complementary XRD analysis confirms the formation of the LDH phase, with basal spacings consistent with Cl^−^ andNO_3_^−^ -intercalated structures [29,30,32]. It should be acknowledged that the focus of the present work is to evaluate the functional antimicrobial response of LDH coatings under application-relevant conditions, rather than to provide a comprehensive metrological characterisation of surface statistics.

Surface topography and chemical composition both reflect on the different wettability behaviour of different LDH formulations. Alongside the different substrate used for different samples (e.g., glass and aluminium sheet), contact angle analysis defines different degrees of surface wettability, although all samples appear hydrophilic. The dynamic wettability behaviour observed in in situ-grown LDH is likely associated with the hierarchical LDH nanoarchitecture. In contrast, etched-glass coatings display stable wetting behaviour over time, consistent with a surface response dominated by the underlying etched substrate. These differences in both static and dynamic wettability reflect the distinct synthesis pathways. LDH deposition on etched-glass results in coatings whose wetting behaviour is primarily influenced by substrate roughness and morphology, whereas in situ growth produces surfaces where wettability is more directly linked to LDH crystal development and the resulting nanostructure.

All the observations arisen from various characterisations and from relevant literature [29,30,31] play a crucial role in the experimental antimicrobial performance of LDH.

Although each physicochemical property of LDHs may contribute individually to their antimicrobial behaviour, the results suggest that the observed antibacterial performance is more likely due to a combined effect of chemical composition, wettability, and surface topography. Chemical composition appears to be a particularly important factor, consistent with previous reports showing that pristine LDHs can exert antimicrobial activity [20,22,26].

The antimicrobial behaviour of LDH coatings is similar between sample type, with ZnAl-based formulations consistently exhibiting the highest activity. Zinc-containing LDHs provide a more inhibitory surface environment, likely through mechanisms affecting membrane integrity and metabolic activity, whereas magnesium-based systems do not exhibit comparable antimicrobial efficacy under the same conditions. The persistence of this trend across both fabrication routes, and across literature [21,23], supports the conclusion that chemical composition is the dominant factor governing antimicrobial performance. One possible explanation is that zinc–bacteria interactions may hinder bacterial proliferation or nutrient uptake through damage to the cell wall [19,21,24,25]. The inefficacy of MgAl-LDH is supported by relevant literature [24].

Surface wettability appears to play a secondary role in modulating the bacterial response, subordinate to the dominant effect of surface chemistry.

Despite ZnAl–LDH coatings on etched-glass samples exhibit a more hydrophilic nature that would generally favour bacterial adhesion, a reduced bacterial proliferation is still observed on such surfaces, indicating that chemical inhibition overrides wettability-driven attachment. Conversely, MgAl-based coatings support bacterial growth despite less favourable wetting conditions, reinforcing the secondary role of wettability in this system.

Topographical effects are present, but they appear to be subordinate to chemical composition. Although etched LDH coatings exhibit heterogeneous surface features due to substrate-induced roughness, similar morphological characteristics across different formulations do not translate into comparable antimicrobial performance. Similarly, SEM observations of in situ-grown LDH indicate that bacterial distribution differs between MgAl- and ZnAl-based coatings, with more localised colonisation around nucleation sites in Mg-based systems and confinement within lamellar structures in Zn-based coatings. This suggests that nanoscale structure alone is insufficient to explain the observed differences in bacterial proliferation. Instead, surface topography appears to act as a modulator of the spatial arrangement and localisation of early biofilm-like structures.

Any contribution to the antimicrobial performance due to synthesis processes can be excluded. For etched LDH coatings, the etched-blank samples exhibit bacterial growth comparable to untreated glass, indicating that any residual species from hydrofluoric acid treatment do not significantly influence bacterial viability. Furthermore, the consistency between etched and in situ LDH results supports the conclusion that antimicrobial activity originates from the LDH coatings rather than from substrate-related artefacts.

Hence, the antimicrobial response of LDH coatings may be understood as the result of a coupled system in which chemical composition, wettability, and nanotopography are inherently interdependent. While the individual contribution of each factor cannot be fully isolated, the consistency of the trends observed across different synthesis approaches and in the relevant literature [18,19,20,21,22,23,25] indicates that zinc-mediated chemical activity is likely the dominant factor. Wettability and nanotopography, rather than acting as primary antimicrobial mechanisms, may therefore modulate bacterial attachment and influence where colonies preferentially localise and develop on the surface, as supported by the SEM analysis.

## 5. Conclusions

In this study, two LDH-coating strategies, deposition onto etched glass and in situ growth, were synthesised and evaluated to investigate whether LDHs can act as passive antimicrobial surfaces, where activity arises from intrinsic physicochemical properties rather than from the release of active agents. The results demonstrate that LDHs can indeed exhibit antimicrobial behaviour in the absence of controlled release mechanisms, with performance primarily governed by their chemical composition. ZnAl-based LDHs significantly reduced CFU mL^−1^ and limited biofilm formation for *E. coli* and *S. aureus*. Importantly, consistent antimicrobial trends were observed across both etched and in situ coatings, despite clear differences in morphology and surface structure. This indicates that the antimicrobial response is an intrinsic property of the LDH material rather than a consequence of fabrication route or substrate effects. While chemical composition defines bacterial viability, surface wettability and nanotopography modulate bacteria–surface interactions. These parameters influence initial adhesion and spatial organisation, such as localisation within lamellar structures or accumulation on exposed features, but do not independently determine antimicrobial performance. Instead, they contribute to a coupled system in which the physicochemical environment at the interface governs bacterial behaviour.

Overall, this work demonstrates that LDHs can function as passive antimicrobial nanostructures, where antibacterial activity emerges from the interplay of intrinsic material properties rather than from the delivery of active agents. This distinction provides a framework for the rational design of stable, long-lasting antimicrobial surfaces that avoid issues associated with leaching, depletion, or regulatory constraints of biocidal additives.

From an application perspective, ZnAl-based LDHs represent promising candidates for antimicrobial coatings in biomedical, food-contact, and marine environments, where durable and non-releasing surfaces are required. More broadly, the ability to tune LDH composition and nanoarchitecture offers opportunities to design surfaces that not only suppress bacterial growth but also control biofilm organisation, opening pathways for both antimicrobial and biofunctional applications.

## Figures and Tables

**Table 1:** Mean contact angle for in situ samples, with and without initial transient response.

Sample Name	In Situ with Transient Response	In Situ Without Transient Response
MgAl-NO_3_	47°	45°
MgAl-Cl_2_	21°	18°
ZnAl-NO_3_	57°	53°
ZnAl-Cl_2_	62°	58°

## Data Availability

Data is available upon request.

## Abbreviations

The following abbreviations are used in this manuscript:

LDH	Layered Double Hydroxides
SA	*Staphylococcus aureus*
EC	*Escherichia coli*
PA	*Pseudomonas aeruginosa*
